# Yellow Fever Outbreak in Eastern Senegal, 2020–2021

**DOI:** 10.3390/v13081475

**Published:** 2021-07-28

**Authors:** Moussa Moïse Diagne, Marie Henriette Dior Ndione, Alioune Gaye, Mamadou Aliou Barry, Diawo Diallo, Amadou Diallo, Lusajo L. Mwakibete, Mamadou Diop, El Hadji Ndiaye, Vida Ahyong, Babacar Diouf, Moufid Mhamadi, Cheikh Tidiane Diagne, Fodé Danfakha, Boly Diop, Oumar Faye, Cheikh Loucoubar, Gamou Fall, Cristina M. Tato, Amadou Alpha Sall, Scott C. Weaver, Mawlouth Diallo, Ousmane Faye

**Affiliations:** 1 Virology Department, Institut Pasteur de Dakar, Dakar 220, Senegal; Marie.NDIONE@pasteur.sn (M.H.D.N.); moufid.mhamadi@ucad.edu.sn (M.M.); cheikhtidiane.DIAGNE@pasteur.sn (C.T.D.); oumar.FAYE@pasteur.sn (O.F.); Gamou.FALL@pasteur.sn (G.F.); Amadou.SALL@pasteur.sn (A.A.S.); Ousmane.FAYE@pasteur.sn (O.F.); 2 Zoology Medical Department, Institut Pasteur de Dakar, Dakar 220, Senegal; Alioune.GAYE@pasteur.sn (A.G.); Diawo.DIALLO@pasteur.sn (D.D.); ElHadji.NDIAYE@pasteur.sn (E.H.N.); Babacar.DIOUF2@pasteur.sn (B.D.); Mawlouth.DIALLO@pasteur.sn (M.D.); 3 Epidemiology, Clinical Research and Data Science Department, Institut Pasteur de Dakar, Dakar 220, Senegal; Aliou.BARRY@pasteur.sn (M.A.B.); Amadou.DIALLO@pasteur.sn (A.D.); Mamadou.DIOP@pasteur.sn (M.D.); Cheikh.LOUCOUBAR@pasteur.sn (C.L.); 4 Chan Zuckerberg Biohub, San Francisco, CA 94158, USA; lusajo.mwakibete@czbiohub.org (L.L.M.); vida.ahyong@czbiohub.org (V.A.); cristina.tato@czbiohub.org (C.M.T.); 5 Kedougou Medical Region, Ministry of Health, Kedougou 26005, Senegal; deffode47@gmail.com; 6 Prevention Department, Ministry of Health, Dakar 220, Senegal; diopboly@yahoo.fr; 7 World Reference Center for Emerging Viruses and Arboviruses, Institute for Human Infections and Immunity and Department of Microbiology and Immunology, University of Texas Medical Branch, Galveston, TX 77555, USA; sweaver@utmb.edu

**Keywords:** yellow fever virus, arbovirus, eastern Senegal, Kedougou, sylvatic lifecycle, virus isolation, next generation sequencing, genotype, lineage, 3′UTR

## Abstract

Yellow fever virus remains a major threat in low resource countries in South America and Africa despite the existence of an effective vaccine. In Senegal and particularly in the eastern part of the country, periodic sylvatic circulation has been demonstrated with varying degrees of impact on populations in perpetual renewal. We report an outbreak that occurred from October 2020 to February 2021 in eastern Senegal, notified and managed through the synergistic effort yellow fever national surveillance implemented by the Senegalese Ministry of Health in collaboration with the World Health Organization, the countrywide 4S network set up by the Ministry of Health, the Institut Pasteur de Dakar, and the surveillance of arboviruses and hemorrhagic fever viruses in human and vector populations implemented since mid 2020 in eastern Senegal. Virological analyses highlighted the implication of sylvatic mosquito species in virus transmission. Genomic analysis showed a close relationship between the circulating strain in eastern Senegal, 2020, and another one from the West African lineage previously detected and sequenced two years ago from an unvaccinated Dutch traveler who visited the Gambia and Senegal before developing signs after returning to Europe. Moreover, genome analysis identified a 6-nucleotide deletion in the variable domain of the 3′UTR with potential impact on the biology of the viral strain that merits further investigations. Integrated surveillance of yellow fever virus but also of other arboviruses of public health interest is crucial in an ecosystem such as eastern Senegal.

## 1. Introduction

Yellow fever virus (YFV) is a mosquito-borne flavivirus (*Flaviviridae* family) first isolated in 1927 from a male patient [1].

Mature virions of 40 nm diameter are icosahedral and comprise a nucleocapsid, composed of capsid (C) protein subunits, surrounded by a lipid bilayer derived from host membranes. The viral envelope is studded with dimers of the envelope (E) glycoprotein and membrane (M) protein [2]. YFV is a positive-sense, single stranded RNA ([+]ssRNA) virus with a genome approximatively 11kb in length [3]. The structural proteins (C, M, and E) and the nonstructural (NS) proteins (NS1, NS2A, NS2B, NS3, NS4A, NS4B, and NS5) are encoded in a single open reading frame (ORF) after production of a polyprotein subsequently processed by proteolytic cleavage [3]. The ORF is flanked by two non-coding ends with a cap-structure at the 5‘ terminus and a very stable stem loop in the 3′ terminus necessary for genome stabilization and initiation of both translation and RNA synthesis [4].

One YFV serotype and seven genotypes have been described in Africa and South America, named West African I, West African II, East African, East/Central African, Angolan, South American I, and South American II [5].

Although many infections are mild, YFV can cause a severe, acute yellow fever (YF) illness with fever, nausea, vomiting, epigastric pain, hepatitis with jaundice, renal failure, hemorrhage, shock, and death in about 47% of cases, while the case-fatality rate for all YFV infections is close to 5% [6].

YFV is endemic in tropical regions of Sub-Saharan Africa and South America, where its maintenance in nature involves a broad spectrum of amplification hosts allowing transmission between non-human primates (NHP), horizontal transmission via competent mosquito vectors (*Haemogogus* and *Aedes* species), and by transovarial transmission in these vectors [7]. Three distinct transmission cycles occur: a sylvatic cycle involving transmission between NHP and mosquitoes, with humans infected through spillover via infected mosquito bites while visiting or working in the forest; an intermediate cycle in the African savannah where mosquito transmission may occur between monkeys and humans living or working in forest border areas; and an urban cycle involving a viremic human amplifiers who contract the virus in either the sylvatic or intermediate cycle, then returns to an urban area where competent urban mosquitoes can then transmit the virus to other humans [7]. In Senegal, YFV is transmitted both in a sylvatic cycle where humans are infected through Aedes mosquito bites in the forest or by *Ae. furcifer* that seek human blood meals within villages [8] and in an urban cycle where YFV is transmitted between humans by the highly anthropophilic mosquito *Ae. aegypti*. Despite the existence of a very effective vaccine, large outbreaks have been reported recently in both South America (Brazil), involving spillover infections, and Africa (Democratic republic of Congo, Angola, Nigeria), involving human amplification and urban vector transmission [9].

The first well-documented YF outbreak in West Africa was described in the 18th century in St-Louis, Senegal [10]. Since then, numerous outbreaks as well as sylvatic circulation have been reported in the country [11,12] despite a limited vaccination campaign to prevent the spread of the virus.

Southeastern Senegal is an enzootic focus of several arboviruses of public health concern where YFV undergoes amplification cycle intervals of approximately 6 years [13]. For instance, human infections were reported in 2011 [14] followed by enzootic circulation noted in 2015 via detection of the virus in sylvatic mosquitoes [8]. A molecular evolution study on sylvatic strains showed that six distinct YFV lineages circulate in Senegal, four of which are found in Kedougou, eastern Senegal, all belonging to the West Africa II genotype [9]. Overall, these past studies highlight the Kedougou region as a key hotspot of YFV.

Here, we describe a recent YF outbreak that occurred in some region of eastern Senegal, and we present the results of the genetic analyzes resulting from the sequencing of the identified strains.

## 2. Materials and Methods

*Arboviruses and Hemorrhagic Fever Viruses surveillance systems in Eastern Senegal*.

### 2.1. Human Surveillance

For human surveillance, three distinct systems detected YFV during this study. Indeed, Senegal has a longstanding YF surveillance system, in the context of the WHO network of YF laboratories where the case definition is an acute onset of fever, with jaundice appearing within 14 days after the onset of the first symptoms [15]. In addition, since 2011, a nationwide Syndromic Sentinel Surveillance Network has been implemented in Senegal (4S network), coordinated by the Ministry of Health (MoH) and Institut Pasteur de Dakar [16]. Moreover and similarly to a previous viro-entomological surveillance program implemented from 2008 to 2013 [17,18], passive surveillance for arbovirus and hemorrhagic fever virus infections has been reactivated in the Kedougou region (12°32′ N, 12°11′ W) since early 2020 among patients presenting with acute febrile illness and attending 4 health facilities in Kedougou and Saraya districts: the Kedougou health district center, the Saraya health district center, the Bandafassi primary health care center and the Kedougou military camp.

After patients from the Kedougou region consented, whole blood samples were collected and sent to the station of the Institut Pasteur de Dakar (IPD) in Kedougou, which ensures the weekly or bi-weekly transfer to IPD in Dakar for laboratory analysis. For the field investigation based on a standard investigation sheet, epidemiological and clinical information was collected with a suspected case defined by the onset malaria-like febrile illness in a resident or visitor of the districts, with at least two of the following symptoms that are not explained by other causes: jaundice, myalgia, arthralgia, abdominal pain, diarrhea, unexplained bleeding and headache. A confirmed YF case was a suspected one with a positive IgM test confirmed by plaque reduction neutralization assays, or a positive PCR test for the targeted virus.

### 2.2. Serology Assays

Recognizing that cross-reactions occur among species of the *Flavivirus* genus and that signs and symptoms of arbovirus infection are not specific, sera were tested by enzyme-linked immunosorbant assay (ELISA) to detect immunoglobulin (Ig)M for YFV and other arboviruses (dengue, West Nile, Zika, chikungunya, Rift Valley Fever and Crimean-Congo Hemorrhagic Fever viruses). Positive samples for any flavivirus were confirmed by plaque reduction neutralization tests (PRNTs) targeting the corresponding virus, as previously described [17].

### 2.3. Molecular Diagnostics

For the real-time RT-qPCR assay, 140 µL of human serum were used for RNA extraction with the QiaAmp Viral RNA Extraction Kit (Qiagen, Heiden, Germany) according to the manufacturer’s protocol. RNA was amplified using a real-time RT-qPCR assay in a one-step format on the ABI 7500 (Applied Biosystems, Foster City, CA, USA) using the Quantitect kit (Qiagen, Hilden, Germany). The 25 µL reaction volume contained 5 µL of extracted RNA, 2× QuantiTect Probe, RT-Master Mix, 10 µM of specific YFV primers and probe with the thermal profile as previously described [19,20].

### 2.4. Mosquito Collections and Processing

Mosquito samples were collected between August and November 2020 in 70 sampling sites including forests, savannah, barren, agriculture, and villages in southeastern Senegal (12°33′25.6″ N, 12°10′41.0″ W) and during a field investigation around YF cases in 3 localities of Kidira (14°27′25″ N, 12°12′49″ W). Host seeking and resting mosquitoes were collected using aspirators, BG-Sentinel-2 traps, and human landing collectors and processed as previously described [8,21,22]. Mosquitoes were sorted into monospecific pools and frozen in liquid nitrogen in the field and transported to IPD for virus isolation attempts.

### 2.5. Virus Isolation and Identification from Mosquito Pools

Mosquito pools were homogenized in 3 mL of L-15 medium (Gibco BRL, Grand Island, NY, USA), using chilled tissue-grinders, supplemented with 20% fetal bovine serum (Gibco) and clarified by centrifugation at 1500× *g*, 4 °C for 10 min as previously described [23]. Following centrifugation, the supernatant was filtered using a 1 mL syringe (Artsana, Como, Italy) and 0.20 μm filters (Sartorius, Göttingen, Germany). After infection of a mosquito C6/36 cell monolayer and incubation for 7–8 days or until the observation of cytopathic effects, the presence of virus was detected by indirect immunofluorescence using an in-house hyper-immune mouse ascitic fluids directed to individual or groups of more than 70 African arboviruses (flaviviruses, bunyaviruses, orbiviruses, and alphaviruses) [24]. This led to a tentative viral identification that was later confirmed by amplification of the virus in the brains of newborn mice to assess the mean survival time followed by viral detection using virus-specific RT-PCR.

### 2.6. Next Generation Sequencing of Yellow Fever Virus

Whole genome targeting sequencing of YFV was undertaken by an amplicon-based approach using manually designed specific multiplex primer pools. YFV genomes from the West Africa region were downloaded from NCBI (List S1). A multiple sequence alignment (MSA) was executed using Geneious Prime 2021.0.3 to attain a consensus genome. Genomes with N% > 1 were excluded from MSA. The following parameters were utilized for the MSA: Cost Matrix = 65% Similarity, Gap open penalty = 12, Gap extension penalty = 3 and refinement iterations = 2. The different primers are available in the Table S1.

Amplicon products were pooled in equal concentration, and libraries were generated using the Illumina DNA preparation kit according to the manufacturer’s specifications. Whole-genome sequencing was performed with paired-end reads using the Illumina MiSeq reagent kit v3 (150 cycles) on an Illumina MiSeq instrument. To generate the consensus genomes, we used the fully open-source EDGE Bioinformatics software [25].

### 2.7. Phylogenetic Analysis

Sequences obtained during this work were submitted to an automated online phylogenetic tool to identify and classify YFV gene sequences [26]. The newly generated YFV genomes were analyzed with other YFV whole genomes selected from different genotypes and lineages available in Genbank. Sequences were aligned using MAFFT [27], and the alignment was run under the best model in IQ-TREE [28]. The maximum-likelihood (ML) phylogenetic tree was visualized using Figtree V1.4.4 [29]. Sequence data were visualized using Snap Gene Viewer V4.3.4 (https://www.snapgene.com/ (accessed on 14 June 2021)).

## 3. Results

### 3.1. First Detected Yellow Fever Human Cases

The first human cases were detected through both the 4S network and the YF national surveillance in Tambacounda (13°46′ N, 13°40′ W) and Matam (15°06′ N, 13°38′ W) medical regions, eastern Senegal (Table 1). A sample from a 40-year-old woman from the Kidira district in Tambacounda medical region suspected to have an arboviral infection was collected on 3 October 2020, and sent to the WHO Collaborating Center of Reference and Research on Arboviruses and Hemorrhagic Fever Viruses in the Virology Department of the Institut Pasteur de Dakar for diagnostic. The laboratory confirmed the case as YF infection on 29 October 2020, after detection of IgM antibodies confirmed by PRNT_90_. On 30 October 2020, a blood sample of an 8-year-old child from Bakel, another city in Tambacounda, presenting symptoms for more than two weeks as well as febrile jaundice, was received for testing by IPD. YFV acute infection was diagnosed with positive results forboth RT-qPCR and ELISA + PRNT assays. On 4 November 2021, a 23-year-old man with fever and jaundice admitted to the regional hospital of Matam was sampled with the molecular and serological assays detecting YFV infection. Like the 8-year-old child, this man succumbed to severe illness. One day later, additional serological evidence of recent YFV infection was made on a sample from a 15-year-old girl from Kidira, who presented herself to the health center with headache. The IPD laboratory also received on 23 November 2021, a sample from a 24-year-old woman from the medical region of Matam who also tested positive by YFV-specific PRNT_90_. Following epidemiological investigations carried out around the latter case, no additional cases or deaths have since been reported in Tambacounda or Matam regions.

### 3.2. Yellow Fever Virus Circulation in the Human Population in Kedougou

The model of arbovirus surveillance in human populations undertaken after 2008 for 5 years was reactivated in mid 2020. Thus, from September 2020 to May 2021, 541 human sera (M/F: 1.84) were received and tested in the virology department of IPD. During this activity, additional YF cases were recorded from early November 2020 to February 2021, 15 with serological evidence and two viremic people. Even though the health district of Kedougou received more than three times the number of cases presented to the health district of Saraya, the percentage of positivity was quite similar with around 3% laboratory-confirmed cases, among them almost half with malarial co-infection (Table 2). Information recorded from the epidemiological investigations showed than about 77% of all the YFV cases declared that they had not left the area within 15 days before the onset of the disease. A brief summary of the YF diagnostics in the context of arbovirus passive surveillance can be found in the Table 2.

### 3.3. Yellow Fever Virus Circulation in Mosquitoes from Kedougou

Only mosquitoes collected in August and September 2020 in Kedougou and November 2020 in Kidira are included in this report. A total of 7474 mosquitoes belonging to 5 genera and more than 41 species were collected. The most abundant *Aedes* species was *Ae. furcifer* (n = 1540; 20.6% of the collected fauna), followed by *Ae. aegypti* (15.5%), and *Ae. vittatus* (11.2%). The other known YFV vectors were *Ae. luteocephalus* (7.5%), *Ae. africanus* (2.2%), and *Ae. taylori* (1.8%). YFV was isolated from 25 mosquito pools belonging to five sylvatic species (Table 3). The frequently infected species was *Aedes* (*Diceromyia*) *furcifer* (13 out of the 25, i.e., 52% of infected pools). Laboratory analyses on the mosquitos trapped in October in Kedougou are ongoing.

### 3.4. Phylogenetic Analyses of the Yellow Fever Virus Strain Causing the Outbreak in Eastern Senegal

**Figure 1:**
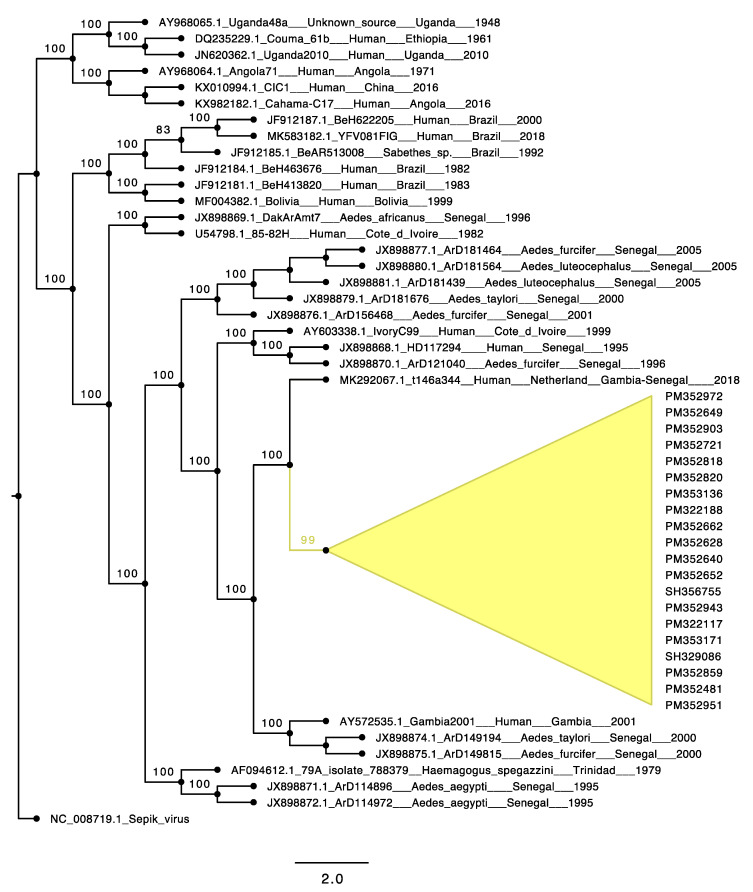
Sepik virus-rooted maximum-likelihood phylogenetic tree of yellow fever virus strains from different genotypes. (Newly sequenced yellow fever viruses are shown in yellow).

Among the four acute human cases and 25 mosquito pools positive for YFV, 20 samples (18 from mosquito pools isolates and 2 from human sera) were selected for Next Generation Sequencing. The details of these samples are available in Table S2. We obtained nearly complete genomic sequences for all samples with 99% nucleotide (nt) identity between the two most divergent within the group. Analysis showed that all of the sequences belong to the West Africa (WA) genotype. ML phylogenies placed these sequences (Genbank accession numbers MZ595182-MZ595199 for mosquitoes and Genbank accession numbers MZ595203-MZ595204 for human) in a single clade falling within lineage 5 of the WA genotype along with other sequences from Kedougou 2000 (Genbank accession numbers JX898874.1 and JX898875.1) and a Gambia 2001 strain (accession number AY572535.1). Moreover, the 2020–2021 YFV eastern Senegal outbreak cluster shared close nt identity with a YFV strain (Figure 1) detected in 2018 from a Dutch traveler who fell ill after returning home following a stay in the Gambia and in Senegal [30].

Genome analysis of the twenty new YFV sequences revealed few non-synonymous mutations in the ORF compared to the sequence detected in the Dutch traveler (Genbank accession number: MK292067.1); three of these were present in all the new sequences, including a switch between two polar amino acids (S487N), a substitution of a polar amino acid with a basic amino acid (Y1725H), and a replacement of a basic amino acid by another one (K2613R). The other non-synonymous mutations within the newly sequenced YFV are detailed in the Table S3.

**Figure 2:**
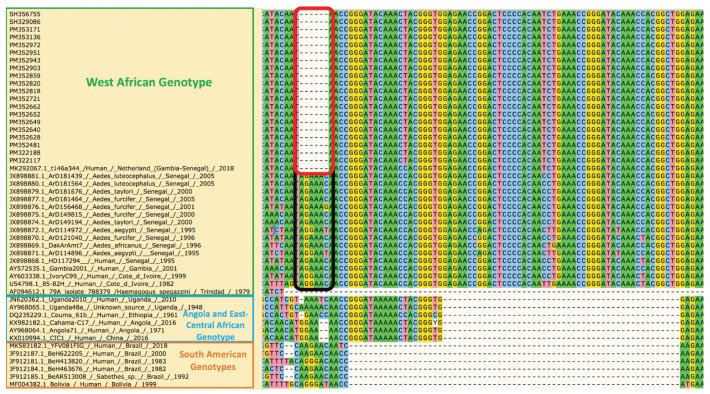
Alignment of portion of the variable domain within the 3′UTR downstream the yellow fever virus polyprotein. (Black band surrounds the first 6 nucleotides downstream the ORF 3′ terminus; red band surrounds the deletion area of newly sequenced strains).

In the partial 3′ genome untranslated region (UTR) obtained, there were 5 mismatches at the nucleotide level: C10383T, 10481delA, G10499A, T10521C, T10549C. Moreover, in all new sequences, we observed a deletion of an *AG(A/G)AAC* sequence present in most 3′UTRs between nucleotides 10368nt and 10373nt of the WA genotype (Figure 2).

## 4. Discussion

We report a YF outbreak that occurred from October 2020 to February 2021 in three different regions of eastern Senegal (Tambacounda, Matam, and Kedougou). The notification of this epidemic and its follow-up involved synergistic efforts of the YF national surveillance implemented by the MoH in collaboration with WHO, the countrywide 4S network set up by the MoH and IPD, and the surveillance of arboviruses and hemorrhagic fever viruses in human and vector populations.

Virological analyses highlighted the implication of sylvatic mosquito species in the virus transmission. Indeed, the high identity observed between the nearly complete genomes of samples from eighteen mosquito pools and two human sera confirmed the circulation of a same YFV strain, belonging to the lineage 5 of the WA genotype, between mosquitoes and human populations in Kedougou, Tambacounda, and potentially in Matam.

Kedougou is a YF-endemic zone, and the virus was mainly found in sylvatic vectors, similar to the previous outbreak in 2010 [24] and 2015 [8]. Then, the epizootic vectors *Ae. luteocephalus* and *Ae. taylori*, as well as the bridge vector *Ae. furcifer*, were found infected by YFV, reinforcing the hypothesis of Diallo et al. [24] that these species comprise the principal vectors of sylvatic YFV in southeastern Senegal. Moreover, the recent outbreak started in October 2020, which is consistent with previous conclusion that the highest risk of human exposure in the region peaks in October, when most of the sites, including villages and agricultural fields, contain YFV-infected mosquitoes [24]. Furthermore, the first detection of YFV in mosquitoes was made in August 2020, i.e., two months before the outbreak notification, reflecting enzootic circulation among sylvatic reservoirs followed by a spillover event.

Although Tambacounda and Matam were the first regions affected by the outbreak, Kedougou recorded the highest number of cases. The relatively recent gold mining activities, forest destruction, increased urbanization, and migration of susceptible populations may be factors leading to increased risk for a YFV outbreak in the region. Regardless, the mass vaccination campaign launched by the Senegalese government in February 2021 was followed by a drastic reduction in cases, with none recorded after the third week of this month, also corresponding to the period of YFV vectors disappearance in this area due to harsh and unfavorable environmental conditions [20].

Genomic analysis showed a close relationship of the etiologic 2020 YF outbreak strains with another detected and sequenced from an unvaccinated Dutch traveler who spent two weeks in the Gambia and then a few days in Senegal in 2018 before developing signs of disease after returning to Europe [28]. At that time, the origin of infection was almost impossible to determine, as there were several reports of YF cases in unvaccinated travelers returning from the Gambia [31,32]. However, our detection of nearly identical sequences among YFV strains from sylvatic mosquitoes in Senegal 2 years later suggests acquisition of infection by the Dutch traveler in Senegal and endemicity of the etiologic strain of the 2020–2021outbreak. Despite the 2–3-year time interval, only few mutations and rarely nonsynonymous mutations accumulated, consistent with previous observations on the genetic stability of YFV [9]. In addition to 3 nonsymonymous mutations, we believe that the 6nt-deletion observed at the 3′UTR could be important. The flaviviral 3′UTR is divided in three domains: a variable domain 1 immediately downstream of the ORF terminus that can be related to adaptation to specific hosts, a moderately conserved domain 2 and highly conserved domain 3 across flavivirus groups [33].

Even if it was previously shown that deletion within the domain 1 could occur due to cell culture passages [34], the actual pattern in the new YFV sequences was also observed in the clinical samples.

Reports that the variable region of the 3′UTR is linked to the virulence are variable. For instance, Sakai et al. showed that deletion in domain 1 increases virulence of tick-borne encephalitis virus [35]. Another study highlighted a positive correlation between the length of 3′UTR deletions and infectivity of the dengue virus serotype 4 [36]. However, Shan et al. reported that 3′UTR deletions attenuate Zika virus replication through diminished viral RNA synthesis [37]. Additionally, the highly conserved part of all flavivirus 3′UTRs includes a virus-derived noncoding RNA called the flaviviral subgenomic RNA (sfRNA) that is responsible for viral pathogenicity, adaptation to hosts, and vector transmissibility [38]. Although the deletion we observed is located upstream of the sfRNA site, (at the stem-loop 2 in the 3′UTR), a potential indirect effect is possible. All of these findings indicate the need to carry out in-depth studies on the biological impact of the 3′UTR YFV deletion we observed.

In summary, YFV continues to be a major public health concern with insufficient vaccine coverage due to a limited vaccine supply, in addition to human demographic trends leading to high numbers of unvaccinated people living in endemic regions [39]. This YFV outbreak in eastern Senegal stresses the importance of routine immunization and vaccination campaigns even during the context of the COVID-19 pandemic. Although some efforts have been made to control YF epidemics at national and international levels, additional support is required in resource-limited areas. The integrated surveillance of YFV but also of other arboviruses of public health importance is crucial in eastern Senegal where the ecosystem is favorable to their maintenance, dissemination, and potential spillovers.

## Figures and Tables

**Table 1:** Summary of the first Yellow Fever cases detected in Tambacounda and Matam. (ct: threshold cycle).

	Age	Sex	Locality	Date of Onset of Symptoms (dd/mm/yyyy)	Sampling Date (dd/mm/yyyy)	Symptoms	YF Vaccination Status	PCR YF	Differencial PCR	IgM YF	Differencial ELISA	PRNT_90_ YF (Titer)	Differencial PRNT_90_ Assays
Patient 1	40	F	Kidira	NA	18/10/2020	Fever	Not vaccinated	-	-	+	-	+ (1/320)	-
Patient 2	8	M	Bakel	13/10/2020	29/10/2020	Not vaccinated	Not vaccinated	+ (ct: 34.9)	-	+	-	+ (1/80)	-
Patient 3	23	M	Kidira	02/11/2020	04/11/2020	Fever + jaundice	Not vaccinated	+ (ct: 33.4)	-	+	-	+ (1/80)	-
Patient 4	15	F	Kidira	NA	02/11/2020	Headache	Not vaccinated	-	-	+	-	+ (1/160)	-
Patient 5	24	F	Matam	NA	19/11/2020	Fever	Not vaccinated	-	-	+	-	+ (1/40)	-

**Table 2:** Summary of YF cases detected in the Kedougou region (* ELISA + PRNT).

Health Districts	No. of Patients	No. of YFV Positive Case (%)	YFV/Malaria Co-Infection (%)	No. of Acute Infection (Positive YFV RT-PCR)	No. of Recently Acquired Infection (Positive YFV Serology Assays *)
Kedougou	421	13 (3.1%)	8 (1.9%)	1	12
Saraya	120	4 (3.3%)	2 (1.7%)	1	3

**Table 3:** Mosquitoes collected and yellow fever virus isolated Summary of the isolation attempts on mosquito pools trapped in eastern Senegal, August-September and November 2020.

Sites	Month	Species	No of Mosquitoes Collected	No of Pools Tested	Infected Species (No Positive Pools)
Kedougou	August	*Aedes aegypti*	661	97	*Ae. furcifer* (6), *Ae. luteocephalus* (3), *Ae. taylori* (1)
		*Ae. africanus*	92	9	
		*Ae. furcifer*	1025	108	
		*Ae. luteocephalus*	419	58	
		*Ae. taylori*	112	33	
		*Ae. vittatus*	495	86	
	September	*Ae. aegypti*	478	65	*Ae. furcifer* (7), *Ae. luteocephalus* (5), *Ae. taylori* (1), *Ae. vittatus* (1), *Ae. africanus* (1)
		*Ae. africanus*	69	11	
		*Ae. furcifer*	515	74	
		*Ae. luteocephalus*	141	33	
		*Ae. taylori*	25	15	
		*Ae. vittatus*	339	64	
Kidira	November	*Ae. aegypti*	19	4	
		*Ae. africanus*	0	0	
		*Ae. furcifer*	0	0	
		*Ae. luteocephalus*	0	0	
		*Ae. taylori*	0	0	
		*Ae. vittatus*	0	0	
All		*Ae. aegypti*	1158	166	*Ae. furcifer* (13), *Ae. luteocephalus* (8), *Ae. taylori* (2), *Ae. vittatus* (1), *Ae. africanus* (1)
		*Ae. africanus*	161	20	
		*Ae. furcifer*	1540	182	
		*Ae. luteocephalus*	560	91	
		*Ae. taylori*	137	48	
		*Ae. vittatus*	834	150	
		others	3084	511	

*Others: Ae. argenteopunctatus, Ae. bromeliae, Ae. centropunctatus, Ae. cumminsii, Ae. dalzieli, Ae. fowleri, Ae. hirsutus, Ae. mcintoshi, Ae. metallicus, Ae. minutus, Ae. ochraceus, Ae. unilineatus, Anopheles brohieri, An. coustani, An. flavicosta, An. funestus, An. gambiae, An. hancocki, An. nili, An. pharoensis, An. rufipes, An. squamosus, Culex antennatus, Cx. bitaeniorhynchus, Cx. cinereus, Cx. ethiopicus, Cx. nebulosus, Cx. perfuscus, Cx. poicilipes, Cx. quinquefasciatus, Cx. sp, Cx. tritaeniorhynchus, Eretmapodites quinquevitattus, Mansonia africana, Ma. Uniformis.*

## Data Availability

Not applicable.

## Supplementary Materials

The following are available online at https://www.mdpi.com/article/10.3390/v13081475/s1. Table S1: Yellow fever virus Primers List. Table S2: Samples from the 2020 YF outbreak in Senegal used for Next Generation Sequencing and their respective Genbank accession numbers. Table S3: Amino acid replacement occurring in the newly sequenced YFV samples compared to the closely related t146a344 (MK292067.1). List S1: Yellow fever virus genomes used for primers design.
