# Supplementary material for: Yellow Fever Outbreak in Eastern Senegal, 2020–2021
Source: Viruses. 2021 Jul 28;13(8):1475. doi: 10.3390/v13081475 (PMC8402698; doi:10.3390/v13081475)
Supplement: Supplementary file 1 [file viruses-13-01475-s001.zip › List S1. YFV_manuscript_Viruses.pdf]

List S1: Yellow Fever virus genomes used for primers design

MN958078, MN211311, MN211310, MN211308, MN211307, MN211306, MN211304,MN211303, MN211302, MK457701, MK292067, MH444798, MF405338, KU978765, AY572535, KU978763, JX898881, JX898880, JX898879, JX898878, JX898877, JX898876, JX898875, JX898874, JX898873, JX898872, JX898871, JX898870, JX898869, JX898868, AY603338.
