# Supplementary material for: Yellow Fever Outbreak in Eastern Senegal, 2020–2021
Source: Viruses. 2021 Jul 28;13(8):1475. doi: 10.3390/v13081475 (PMC8402698; doi:10.3390/v13081475)
Supplement: Supplementary file 1 [file viruses-13-01475-s001.zip › Table S1. YFV_manuscript_Viruses.pdf]

Table S1. Yellow Fever virus Primers List

Primer	Primer Sequence
FP01A	AGTAAATCCTGTGTGCTAATTGAGG
FP05A	AGAGGCTGGGGYAATGGYTGT
FP09A	TCAGTTGACTCCCTTGARCATGA
FP13A	GGCCTGTGGAAGTAYCTRAATG
FP17A	GATTTTCATCCTGGAGCTGGG
FP21A	GGTGGAGAGGCAATGGAYA
FP25A	GCTGGTGTTACTAYGCTGCT
FP29A	CTGCAATGGATGGTGGTGGA
RP02B	TCATCTGGCTCCTCTCTTGG
RP06B	CACTTCATCATCATTRGTTGARGCK
RP10B	CGTCCRTCRCAGTTGCCAT
RP14B	ACATCCCCACTTCTCCTAGC
RP18B	TGCCACCTTCCTCCCTTCAT
RP22B	TGTGCAAGCTTTGACTGCTGC
RP27A	TTGGCAGTCTTCCACTGTTC
RP30B	CACTCCCCTTTCCCATGAATC
FP02B	AGACCTGGACCYTCAAGAGG
FP07A	CCAGTGATAGTAGCTGATGAYCT
FP10B	TTCCAGATAGAGGAGTTTGGGA
FP14B	GCAACCCGCMTATTTGGGC

FP19A	ATGGGAGCCAACCTWTGYG
FP23A	CAYGCTCCACTGGTCTCTY
FP27A	AAAATCTGAGTACATGACCWCYTGG
FP31A	CTCATGGGTYCCACAAGGA
RP04B	CACAAGTGAATTTGGCGCATG
RP08B	GCATTGATCTCATCTGCCCTRC
RP12B	TTGATGCTTTCCTAGAAGCAACAG
RP16B	GATCTGTGGGAGRAAMCGTCTT
RP20B	ATTGCGGTAAGCCCTAGAGC
RP24B	GTTTCTCATGGCCGTCTCTTC
RP28B	TCCCATCCAGCGGTGTCAT
RP33A	AGTGGTTTTGTGTTTGTCATCCAAA
