# Supplementary material for: Yellow Fever Outbreak in Eastern Senegal, 2020–2021
Source: Viruses. 2021 Jul 28;13(8):1475. doi: 10.3390/v13081475 (PMC8402698; doi:10.3390/v13081475)
Supplement: Supplementary file 1 [file viruses-13-01475-s001.zip › Table S2. YFV_manuscript_Viruses_Revised.pdf]

Table S2. Samples from the 2020 YF outbreak in Senegal used for Next Generation Sequencing and their respective Genbank accession numbers**.**

ID	Collection date	Year	Location	Genius	Sex	No mosquitos	Age (year)	Sample type	Genbank reference number
PM322117	August	2020	Kédougou	*Aedes taylori*	Females	3	NA	Isolate	MZ595182
PM322188	August	2020	Kédougou	*Aedes luteocephalus*	Females	63	NA	Isolate	MZ595183
PM352481	August	2020	Kédougou	*Aedes luteocephalus*	Females	34	NA	Isolate	MZ595184
PM352628	August	2020	Kédougou	*Aedes furcifer*	Females	20	NA	Isolate	MZ595185
PM352640	August	2020	Kédougou	*Aedes furcifer*	Females	23	NA	Isolate	MZ595186
PM352649	August	2020	Kédougou	*Aedes furcifer*	Females	36	NA	Isolate	MZ595187
PM352652	August	2020	Kédougou	*Aedes luteocephalus*	Females	18	NA	Isolate	MZ595188
PM352662	August	2020	Kédougou	*Aedes furcifer*	Females	5	NA	Isolate	MZ595189
PM352721	August	2020	Kédougou	*Aedes furcifer*	Females	61	NA	Isolate	MZ595190
PM352818	September	2020	Kedougou	*Aedes furcifer*	Females	4	NA	Isolate	MZ595191
PM352820	September	2020	Kedougou	*Aedes luteocephalus*	Females	23	NA	Isolate	MZ595192
PM352859	September	2020	Kedougou	*Aedes luteocephalus*	Females	5	NA	Isolate	MZ595193
PM352903	September	2020	Kedougou	*Aedes furcifer*	Females	26	NA	Isolate	MZ595194
PM352943	September	2020	Kedougou	*Aedes furcifer*	Females	3	NA	Isolate	MZ595195
PM352951	September	2020	Kedougou	*Aedes furcifer*	Females	7	NA	Isolate	MZ595196
PM352972	September	2020	Kedougou	*Aedes furcifer*	Females	21	NA	Isolate	MZ595197
PM353136	September	2020	Kedougou	*Aedes luteocephalus*	Females	15	NA	Isolate	MZ595198
PM353171	September	2020	Kedougou	*Aedes furcifer*	Females	3	NA	Isolate	MZ595199
SH329086	October	2020	Bakel	Human	Male	NA	8	Clinical	MZ595203
SH356755	November	2020	Kedougou	Human	Male	NA	10	Clinical	MZ595204
