# Supplementary material for: Yellow Fever Outbreak in Eastern Senegal, 2020–2021
Source: Viruses. 2021 Jul 28;13(8):1475. doi: 10.3390/v13081475 (PMC8402698; doi:10.3390/v13081475)
Supplement: Supplementary file 1 [file viruses-13-01475-s001.zip › Table S3. YFV_manuscript_Viruses.pdf]

Table S3. Amino acid replacement occuring in the the newly sequenced YFV samples compared to the closely related t146a344 (MK292067.1)

Amino Acid mutations	Samples
T721M	PM352640
F978S	PM352859
T1185I	PM352481 and PM352951
F1300I	PM322117 and PM353171
L1350I	SH329086
L1468F	PM352481 and PM352951
K1662E	PM352481
G2127S	PM352640
K2273R	PM352943 and SH356755
Y2488H	PM352859
K2502E	PM352943
H3067Y	PM352943
